# Alzheimer’s Disease, Hearing Loss, and Deviance Detection

**DOI:** 10.3389/fnins.2022.879480

**Published:** 2022-06-02

**Authors:** David Pérez-González, Thomas G. Schreiner, Daniel A. Llano, Manuel S. Malmierca

**Affiliations:** ^1^Cognitive and Auditory Neuroscience Laboratory (Lab 1), Institute of Neuroscience of Castilla y León (INCYL), University of Salamanca, Salamanca, Spain; ^2^Institute for Biomedical Research of Salamanca (IBSAL), Salamanca, Spain; ^3^Department of Electrical Measurements and Materials, Faculty of Electrical Engineering and Information Technology, “Gheorghe Asachi” Technical University of Iasi, Iaşi, Romania; ^4^Department of Neurology, “Gr. T. Popa” University of Medicine and Pharmacy, Iaşi, Romania; ^5^Department of Molecular and Integrative Physiology, University of Illinois at Urbana-Champaign, Champaign, IL, United States; ^6^The Beckman Institute for Advanced Science and Technology, Urbana, IL, United States; ^7^Carle Neuroscience Institute, Urbana, IL, United States; ^8^Department of Cell Biology and Pathology, Faculty of Medicine, University of Salamanca, Salamanca, Spain

**Keywords:** neurodegeneration, cognitive deficits, predictive coding, acetylcholine, cholinergic, stimulus-specific adaptation

## Abstract

Age-related hearing loss is a widespread condition among the elderly, affecting communication and social participation. Given its high incidence, it is not unusual that individuals suffering from age-related hearing loss also suffer from other age-related neurodegenerative diseases, a scenario which severely impacts their quality of life. Furthermore, recent studies have identified hearing loss as a relevant risk factor for the development of dementia due to Alzheimer’s disease, although the underlying associations are still unclear. In order to cope with the continuous flow of auditory information, the brain needs to separate repetitive sounds from rare, unexpected sounds, which may be relevant. This process, known as deviance detection, is a key component of the sensory perception theory of predictive coding. According to this framework, the brain would use the available incoming information to make predictions about the environment and signal the unexpected stimuli that break those predictions. Such a system can be easily impaired by the distortion of auditory information processing that accompanies hearing loss. Changes in cholinergic neuromodulation have been found to alter auditory deviance detection both in humans and animal models. Interestingly, some theories propose a role for acetylcholine in the development of Alzheimer’s disease, the most common type of dementia. Acetylcholine is involved in multiple neurobiological processes such as attention, learning, memory, arousal, sleep and/or cognitive reinforcement, and has direct influence on the auditory system at the levels of the inferior colliculus and auditory cortex. Here we comment on the possible links between acetylcholine, hearing loss, and Alzheimer’s disease, and association that is worth further investigation.

Age-related hearing loss is the most prevalent disability/disease-related condition in the world (World Health Organization [WHO], 2004). Disabling hearing loss affects an estimated 430 million people worldwide, according to a recent report by the World Health Organization (World Health Organization [WHO], 2021). This disorder is particularly prominent in elderly people, with a prevalence of 65% of people aged 60 years or more suffering hearing loss in any degree, and 25% of people in this age group suffering moderate or severe hearing loss. Importantly, hearing loss leads to a significant reduction in quality of life and social isolation (Lin and Albert, 2014).

Even moderate age-related hearing loss causes deficits of speech understanding in many otherwise cognitively intact, aging people, and subsequently, many of these individuals withdraw from active participation in society. Moreover, this problem is epidemiologically associated with cognitive impairments that typically affect the elderly, such as dementia due to Alzheimer’s disease (AD), to the point that there may exist a causal relationship even when ruling out the effect of age. This scenario profoundly influences the quality of life of these individuals and their families and causes a substantial impact on social welfare and health system costs. Given the lack of established disease-modifying treatments for any neurodegenerative cause of dementia, a better knowledge of the mechanisms of the alterations that occur in these neurodegenerative pathologies is necessary to prevent their occurrence, and thus it has become a world priority. Indeed, in a seminal work launched in 2017, the Lancet’s “Commission on dementia prevention, intervention, and care” (Livingston et al., 2017) proposed a model, in which hearing loss emerged as the most important and modifiable mid-life risk factor for the development of dementia. This finding undoubtedly has important implications for the understanding of dementia.

The links between hearing loss and dementia are incompletely elucidated and are under debate (Griffiths et al., 2020; Nadhimi and Llano, 2021). Despite the fact that there are numerous risk factors shared by the two pathologies, the causal element in humans remains to be discovered. In recent years, there has been an increasing number of epidemiological studies that have suggested this association. In a meta-analysis of 36 observational studies, Loughrey et al. (2018) found significant associations between age related hearing loss and a series of cognitive impairments and dementia. Ford et al. (2018) found the association valid in men according to which, males suffering hearing loss were more likely to develop dementia, an idea confirmed and supported also by the meta-analysis performed by the authors. Similarly, an association between hearing impairment with increased prevalence of dementia has been described in several studies (Lin et al., 2011a,b; Heywood et al., 2017).

In a recent detailed study, Griffiths et al. (2020) proposed several potential mechanisms linking hearing loss and more general cognitive impairment. First, a shared microvascular pathology at the levels of the peripheral hearing apparatus and brain could cause a common pathology producing both hearing loss and dementia. Second, the decreased stimulation caused by a reduced auditory input could produce neuronal changes at multiple levels that could affect cognition negatively. Third, since hearing loss makes listening more difficult, listening could require more cognitive resources, that would not be available for other cognitive tasks. Finally, there could even be direct interactions between changes in cognitive auditory functions and AD pathology. Additionally, Nadhimi and Llano (2021) propose that noise exposure could produce a toxic milieu inducing both short- and long-term changes in the hippocampus.

There are many different types of dementia, and AD is the most common, followed by vascular dementia and dementia with Lewy bodies. Mixed dementia with features of more than one cause is also common, as well as frontotemporal degeneration and dementias associated with brain injury, infections, and alcohol abuse, although less widespread. Cognitive deficits in neurodegenerative diseases have often been characterized as the loss of core functional modules in distinct brain regions, such as “memory centers” or “executive centers.” This classical approach emphasizes the functional difference between disorders, at a time when preclinical models suggest convergence in the pathophysiology of different diseases (Kocagoncu et al., 2020). Further, recent work suggests that aging leads to decreased weighting of sensory inputs and increased dependence on sensory predictions (Lin and Albert, 2014) suggesting that a core cognitive deficit in aging and dementia may involve inappropriate integration of bottom-up and top-down signals.

Sound plays an essential role in human life, starting from basic processes of survival (e.g., a car approaching from behind) to many human activities (including verbal communication or the joy of music). Therefore, the ability to hear and properly recognize sounds is critical to human perception. Humans, like other animals, live with the constant and often overwhelming flow of sensory information coming from the environment. For perception of behaviorally relevant stimuli to occur, sensory systems are organized to identify information sources efficiently. This is true in the auditory system, which is highly complex anatomically and physiologically (Malmierca et al., 1993, 1998; Bajo et al., 1999; Malmierca, 2003, 2004, 2015). In particular, the auditory system needs to be able to distinguish between repetitive, irrelevant sounds (such as the monotonous hum of traffic while driving), and rare or unpredictable sounds that provide new information about the environment (such as the screech of braking tires or a car horn blast) that are relevant for survival. This ability to detect unexpected sounds that violate the regularities established by previous stimuli is usually referred to as *deviance detection*.

According to the *predictive coding* theory (Friston, 2005), the brain constantly generates top-down predictions that are compared with sensory bottom-up signals. The responses to stimuli that match predictions are suppressed, whereas unexpected stimuli that do not match the predictions generate an error signal (enhanced response). These prediction errors are forwarded to the higher level, where they are used to update the internal representation model and thus generate new predictions. In other words, sensory information is continuously shared between low sensory input levels, and higher levels that provide predictions about future sensory input. The canonical microcircuits for predictive coding use feedforward (i.e., bottom-up) connections to convey prediction errors while feedback (i.e., top-down) connections convey predictions (Bastos et al., 2012), and there is a growing body of recent data on the cellular basis of predictive coding in animal models (Malmierca et al., 2009, 2019; Antunes and Malmierca, 2011, 2014; Duque et al., 2012, 2016; Parras et al., 2017, 2021; Carbajal and Malmierca, 2018, 2020; Casado-Román et al., 2020).

A further yet very important and critical implication for predictive coding is directly related to hearing loss and its restoration. The numerous descending projections found in the auditory system reveal the importance of neural feedback pathways, but it is unclear how cognitive processes from higher levels may affect purely sensory processes at the lowest levels. If that were the case, hearing implants could benefit from using neural input from higher auditory regions. And vice versa, it is also unclear whether partial sensory restoration, as currently produced by auditory implants, offers the kind of information required for efficient predictive coding function downstream. For brainstem and cortical implants, an understanding of predictive neural processes is of even greater importance, as predictive processes may underlie the systems these implants are intended to replace. Thus, it is also extremely relevant to find out how AD and dementia are influenced by age-related hearing loss, and whether AD patients could benefit from an improved restoration of hearing that preserves, or at least takes into account, predictive processes. Understanding this association would allow not only the development of strategies for prevention, detection and treatment of age-related hearing loss, but also contextualizing it in relation to its possible impact on the natural course of dementia.

Aging typically degrades the precision of peripheral and central processing, which leads to decreased weighting of sensory inputs and increased reliance on predictions (Wolpe et al., 2016; Chan et al., 2021). Recent neurocomputational research quantifying the synaptic coupling underlying mismatch negativity (MMN, a scalp-recorded auditory evoked potential, related to deviance detection) also found an age-related attenuation of learning-dependent increase in forward connectivity from primary auditory cortex suggesting a reduced sensitivity to the ascending prediction errors (Moran et al., 2014) as well as an age-related increase of the inhibitory effect at inferior frontal gyrus, indicating increased firing rate of the inhibitory neurons (Cooray et al., 2014) over the lifespan. It seems that older brains are less predisposed to updating the prior probability estimate, leading to a perception of the environment increasingly dominated by top-down information. In other words, age turns our brain into a stubborn prediction machine where the sensory input is underrated. This model is consistent with reports on age-related shifts in neuronal recruitment from sensory to frontal regions during sensory processing (Davis et al., 2008).

The reliance on auditory predictions can be significantly disrupted in mild cognitive impairment and dementia. These abilities use temporo-parietal areas that are affected by AD (Golden et al., 2015), and accordingly, patients have difficulty using top-down information to follow conversations in the presence of background noise. Patients with AD show impairments in segregating, tracking and grouping auditory objects that evolve over time (Goll et al., 2012), and in perceiving sound location and motion (Golden et al., 2015). They are also worse at adapting to expected auditory stimuli as they show reduced auditory MMN responses (Pekkonen et al., 2001; Laptinskaya et al., 2018). Even otherwise healthy APOE4 carriers (i.e., elevated risk of AD) show impairments in detecting auditory targets using contextual information (Zimmermann et al., 2019). Patients with amnestic and logopenic phenotypes of AD are impaired in processing a melodic contour, which depends on working memory to predict the upcoming sounds (Golden et al., 2017). All these ideas are highlighted by Kocagoncu et al. (2020), and reviewed by Swords et al. (2018).

One of the leading theories trying to explain the pathophysiology of AD points to the neurotoxic effects of the aggregates of amyloid beta peptide, which, interestingly, are associated with cholinergic system dysfunction from the earliest stages of the disease. Clinically, the symptomatology of AD in which memory disorders predominate is associated with the dysfunction (up to destruction) of the synapses of cholinergic neurons in the hippocampus or nucleus basalis of Meynert (Hampel et al., 2018). There is a direct correlation between altered cholinergic synaptic transmission and cognitive deficit in AD models (Zhu et al., 2017; Bekdash, 2021). Moreover, it has been demonstrated that choline acetyltransferase (ChAT) enzyme transcription is severely diminished in the remaining cholinergic neurons, which leads to decreased ChAT activity and progression of dementia (Hampel et al., 2018). Another enzyme, acetylcholinesterase (AChE), has been shown to be involved in the interaction with the amyloid beta peptide, promoting its aggregation and plaque/fibril formation (Carvajal and Inestrosa, 2011). Furthermore, a recent PET study found that AD patients show reduced levels of the vesicular ACh transporter (a glycoprotein responsible for loading ACh into the synaptic vesicles) in temporal–parietal cortex, the posterior portions of the cingulate gyri and the medial and lateral frontal cortex (Aghourian et al., 2017). At the same time, in the hippocampus (a strongly affected area in AD), a reduction in the expression of muscarinic and nicotinic cholinergic receptors was observed (Jiang et al., 2014; Lombardo and Maskos, 2015). In practice, the use of anticholinesterase medication (inhibitors such as Donepezil, Rivastigmine) with partially favorable effect brings an additional argument in the importance of acetylcholine (ACh) as a piece in the complex puzzle of AD pathophysiology (Sharma, 2019).

ACh is a widely distributed neuromodulator throughout the brain, including the inferior colliculus (IC) and the auditory cortex (AC). Neuromodulation by ACh has been found to have a direct effect on the deviance detection processing carried out by neurons located in the IC, a midbrain auditory station where almost all ascending auditory information converges before progressing onto the auditory thalamus and, ultimately, the AC. The IC is the first stage in the ascending auditory pathway to show stimulus-specific adaptation (SSA), a neuronal phenomenon proposed to contribute to the generation of deviance detection at a cellular level (Pérez-González et al., 2005; Malmierca et al., 2009; Pérez-González and Malmierca, 2014). SSA in the IC is known to be modulated by multiple neurotransmitters and neuromodulators, including glutamate, GABA, cannabinoids, dopamine and also ACh (Pérez-González et al., 2012; Ayala and Malmierca, 2015; Ayala et al., 2016; Valdés-Baizabal et al., 2017, 2020; Carbajal et al., 2020). Applying agonists and antagonists of cholinergic receptors by microiontophoresis, Ayala and Malmierca (2015) found that the activation of IC cholinergic receptors reduced SSA, and that effect was mediated mainly by muscarinic receptors.

In the AC, ACh modulates different neurobiological processes such as attention, learning, memory, arousal, sleep and/or cognitive reinforcement (Dalley et al., 2004; Franklin and Frank, 2015; Batista-Brito et al., 2018). The main source of ACh to the AC is the basal forebrain (Zaborszky et al., 2008; Mesulam, 2013; Chavez and Zaborszky, 2017). In the auditory system, cholinergic modulation is known to alter frequency response areas generating changes across frequency tuning, decreasing the acoustic threshold at the characteristic frequency and changing the encoding of spectral representation of many auditory neurons (Ma and Suga, 2005; Metherate, 2011). Thus, ACh promotes neuronal and synaptic plasticity at different temporal scales (Kilgard and Merzenich, 1998; Kamke et al., 2005). ACh can directly affect the responses of pyramidal neurons in AC, boosting response gain (Noudoost and Moore, 2011; Wood et al., 2017; Batista-Brito et al., 2018). ACh can also indirectly disinhibit pyramidal neurons through the activation of vasoactive intestinal peptide (VIP) interneurons, which express cholinergic receptors (Tremblay et al., 2016); VIP interneurons strongly inhibit somatostatin (SST) interneurons, that in turn inhibit pyramidal neurons. Yet another possibility is that ACh can directly activate SST interneurons, which inhibit parvalbumin (PV) interneurons (Xu et al., 2013), producing a similar disinhibitory effect on pyramidal neurons. In addition, it has been shown that optogenetic photosuppression of PV-mediated inhibition in AC leads to a non-specific increase of neuronal responses, enhancing equally the responses to deviant and standard tones, while similar optogenetic photosuppression of SST-mediated inhibition selectively reduces excitatory responses to frequent tones (Natan et al., 2015). Moreover, long-lasting habituation involves a selective increase in SST-mediated inhibition (Kato et al., 2015). Both putative excitatory and inhibitory neurons across the AC show deviance detection properties, and only in the primary AC deviance detection seems to be more prominent in inhibitory rather than excitatory neurons (Pérez-González et al., 2021). Thus, there are multiple opportunities by which ACh can modulate deviance detection, acting through several mechanisms and microcircuits involving VIP, SST, and PV inhibitory interneurons.

Neuromodulatory inputs such as ACh can shift the relative contribution of bottom-up and top-down signals influencing prediction errors. But how different levels of modulatory inputs alter the sensitivity by which brain circuits prioritize and respond to sensory information or report prediction errors remains unclear. AD is characterized by declines in brain cholinergic neurons (e.g., Coyle et al., 1983), and cognitive decline in AD can be treated by elevating synaptic ACh levels. Thus, the decline in salience signaling (due to lowered ACh), coupled with declines in peripheral hearing in AD, may provide a pathological perfect storm to disrupt predictive processing in AD, contributing to the cognitive deficits seen in this disorder.

In conclusion, there is increasing evidence in the literature of links between Alzheimer’s disease, hearing loss and deviance detection. How are they related to each other is a topic that deserves to be further explored, and potentially could shed light on some basic features of brain function, as well as on the consequences suffered by AD patients. Taken together, the studies mentioned in this Perspective suggest that a core cognitive deficit in AD may involve a deficiency of predictive coding, and this deficiency may be related to the loss of cholinergic adjustment of weighting of bottom-up and top-down inputs in conjunction with loss of auditory sensory input. To better understand the relationship between hearing loss and AD, future studies should examine whether modulation of peripheral hearing and/or cholinergic tone alters auditory predictive behavior or cognitive function more generally. While the alterations related to the cholinergic system seem to play common roles in both hearing loss and AD pathogeny, other intricate mechanisms could also be involved. Amyloid beta toxicity, oxidative stress, and chronic inflammation could be also relevant for explaining this association between two pathologies with significant impact in humans and are worth to be studied in the future.

## Data Availability Statement

The original contributions presented in the study are included in the article/supplementary material, further inquiries can be directed to the corresponding author/s.

## Author Contributions

DP-G, TS, DL, and MM wrote the manuscript. All authors contributed to the article and approved the submitted version.

## Conflict of Interest

The authors declare that the research was conducted in the absence of any commercial or financial relationships that could be construed as a potential conflict of interest.

## Publisher’s Note

All claims expressed in this article are solely those of the authors and do not necessarily represent those of their affiliated organizations, or those of the publisher, the editors and the reviewers. Any product that may be evaluated in this article, or claim that may be made by its manufacturer, is not guaranteed or endorsed by the publisher.
